# Estimation of maternal and child mortality one year after user-fee elimination: an impact evaluation and modelling study in Burkina Faso

**DOI:** 10.2471/BLT.13.130609

**Published:** 2014-09-03

**Authors:** Mira Johri, Valéry Ridde, Rolf Heinmüller, Slim Haddad

**Affiliations:** a Centre de Recherche du Centre Hospitalier de l’Université de Montréal, Tour Saint-Antoine (Porte S03-458), 850 rue St-Denis, Montréal, Québec, H2X 0A9, Canada.; b Institut de Recherche en Sciences de la Santé, Centre national de la recherche scientifique et technologique du Burkina Faso, Ouagadougou, Burkina Faso.

## Abstract

**Objective:**

To estimate the impact on maternal and child mortality after eliminating user fees for pregnant women and for children less than five years of age in Burkina Faso.

**Methods:**

Two health districts in the Sahel region eliminated user fees for facility deliveries and curative consultations for children in September 2008. To compare health-care coverage before and after this change, we used interrupted time series, propensity scores and three independent data sources. Coverage changes were assessed for four variables: women giving birth at a health facility, and children aged 1 to 59 months receiving oral rehydration salts for diarrhoea, antibiotics for pneumonia and artemesinin for malaria. We modelled the mortality impact of coverage changes in the Lives Saved Tool using several scenarios.

**Findings:**

Coverage increased for all variables, however, the increase was not statistically significant for antibiotics for pneumonia. For estimated mortality impact, the intervention saved approximately 593 (estimate range 168–1060) children’s lives in both districts during the first year. This lowered the estimated under-five mortality rate from 235 deaths per 1000 live births in 2008 to 210 (estimate range 189–228) in 2009. If a similar intervention were to be introduced nationwide, 14 000 to 19 000 (estimate range 4000–28 000) children's lives could be saved annually. Maternal mortality showed a modest decrease in all scenarios.

**Conclusion:**

In this setting, eliminating user fees increased use of health services and may have contributed to reduced child mortality.

## Résumé

**Objectif:**

Estimer l'impact sur la mortalité maternelle et la mortalité infantile après la suppression du ticket modérateur pour les femmes enceintes et les enfants âgés de moins de cinq ans au Burkina Faso.

**Méthodes:**

Deux districts sanitaires dans la région du Sahel ont supprimé le ticket modérateur pour les accouchements et les consultations curatives pour les enfants en septembre 2008. Afin de comparer la couverture en matière des soins de santé avant et après ce changement, nous avons utilisé des séries chronologiques interrompues, des scores de propensions et trois sources indépendantes de données. Les changements de couverture ont été évalués pour 4 variables: les femmes accouchant dans un établissement de santé et les enfants âgés de 1 à 59 mois recevant des sels de réhydratation par voie orale pour la diarrhée, des antibiotiques pour la pneumonie et de l'artémisinine pour le paludisme. Nous avons modélisé l'impact des changements de la couverture sur la mortalité dans « Lives Saved Tool » en utilisant plusieurs scénarios.

**Résultats:**

La couverture a augmenté pour toutes les variables; cependant, l'augmentation n'était pas statistiquement significative pour les antibiotiques administrés pour traiter la pneumonie. En ce qui concerne l'impact estimé sur la mortalité, l'intervention a sauvé approximativement 593 vies d'enfants (fourchette d'estimation: 168–1060) dans les 2 districts pendant la première année. Cela a diminué le taux estimé de mortalité des enfants de moins de 5 ans, de 235 décès pour 1000 naissances vivantes en 2008 à 210 décès pour 1000 naissances vivantes (fourchette d'estimation: 189–228) en 2009. Si une action similaire était lancée à l'échelle nationale, cela pourrait sauver 14 000 à 19 000 vies d'enfants (fourchette d'estimation: 4000–28 000) annuellement. La mortalité maternelle n'a montré qu'une diminution modeste dans tous les scénarios.

**Conclusion:**

Dans cette région, la suppression du ticket modérateur a augmenté l'utilisation des services de santé et a peut-être contribué à la réduction de la mortalité infantile.

## Resumen

**Objetivo:**

Estimar el impacto en la mortalidad materna e infantil después de la eliminación de las cuotas de los usuarios para las mujeres embarazadas y para los niños menores de cinco años de edad en Burkina Faso.

**Métodos:**

En septiembre de 2008, dos distritos de salud en la región del Sahel eliminaron las cuotas para los usuarios en las maternidades y las consultas curativas para niños. A fin de comparar la cobertura sanitaria antes y después de este cambio, se utilizaron series de tiempo interrumpido, puntuaciones de propensión y tres fuentes de datos independientes. Se evaluaron cuatro variables en los cambios en la cobertura: mujeres que dieron a luz en un centro de salud y niños de 1 a 59 meses que recibieron sales de rehidratación oral para la diarrea, antibióticos para la neumonía y artemisinina para la malaria. Expusimos el impacto en la mortalidad de los cambios de cobertura en la herramienta "Lives Saved Tool" empleando varios escenarios.

**Resultados:**

La cobertura aumentó para todas las variables, sin embargo, el aumento no fue estadísticamente significativo en el caso de los antibióticos para la neumonía. Por impacto de la mortalidad estimado, la intervención salvó aproximadamente la vida de 593 niños (rango estimado 168 - 1060) en ambos distritos durante el primer año. Esto redujo la tasa estimada de mortalidad de menores de cinco años de 235 muertes por cada 1000 nacidos vivos en 2008 a 210 (rango estimado 189–228) en 2009. Una intervención similar puesta en práctica a nivel nacional podría salvar la vida a entre 14 000 y 19 000 niños al año (rango estimado 4000–28 000). La mortalidad materna mostró una disminución modesta en todos los escenarios.

**Conclusión:**

En esta configuración, la eliminación de las cuotas para los usuarios aumentó la utilización se los servicios sanitarios y pudo haber contribuido a reducir la mortalidad infantil.

## ملخص

**الغرض:**

تقدير الأثر على معدل وفيات الأمهات والأطفال بعد إلغاء الرسوم المفروضة على المستخدمين بالنسبة للنساء الحوامل والأطفال الأقل من خمس سنوات في بوركينا فاسو.

**الطريقة:**

ألغت منطقتان صحيتان في إقليم الساحل الرسوم المفروضة على المستخدمين من أجل إيتاء المرافق والمشاورات العلاجية للأطفال في أيلول/سبتمبر 2008. ولمقارنة تغطية الرعاية الصحية قبل هذا التغير وبعده، استخدمنا السلاسل الزمنية المتقطعة ودرجات القابلية وثلاثة مصادر مستقلة للبيانات. وتم تقييم التغيرات في التغطية للتوصل إلى أربعة متغيرات: النساء اللاتي يلدن في مرفق صحي، والأطفال الذين تتراوح أعمارهم من شهر إلى 59 شهراً الذين يتلقون أملاح الإماهة الفموية لعلاج الإسهال، والمضادات الحيوية لعلاج الالتهاب الرئوي، والأرتيميسينين لعلاج الملاريا. وقمنا بنمذجة أثر التغيرات في التغطية على معدل الوفيات في أداة الأرواح التي تم إنقاذها باستخدام العديد من السيناريوهات.

**النتائج:**

ازدادت التغطية بالنسبة لجميع المتغيرات، ولكن لم تكن الزيادة كبيرة من الناحية الإحصائية بالنسبة لمتغير المضادات الحيوية لعلاج الالتهاب الرئوي. وبالنسبة للأثر على معدل الوفيات المقدر، أدى التدخل إلى إنقاذ أرواح 593 طفلاً تقريباً (نطاق التقدير من 168 إلى 1060) في كلتا المنطقتين خلال السنة الأولى. وأدى هذا إلى خفض معدل الوفيات المقدر للأطفال دون سن الخامسة من 235 وفاة لكل 1000 وليد حي في 2008 إلى 210 وفاة (نطاق التقدير من 189 إلى 228) في 2009. وفي حالة تقديم تدخل مشابه على الصعيد الوطني، من الممكن إنقاذ أرواح من 14000 إلى 19000 طفل (نطاق التقدير من 4000 إلى 28000) سنوياً. وأظهر معدل وفيات الأمهات انخفاضاً متواضعاً في جميع السيناريوهات.

**الاستنتاج:**

أدى إلغاء الرسوم المفروضة على المستخدمين في هذه البيئة إلى زيادة استخدام الخدمات الصحية وربما أسهم في خفض معدل وفيات الأطفال.

## 摘要

**目的:**

估计布基纳法索消除孕妇和未满五岁儿童用户费用后对孕产妇和儿童死亡率的影响。

**方法:**

2008年9月萨赫勒地区两个卫生区取消了用户住院分娩和儿童治疗咨询费用。为比较此变更前后医疗覆盖情况，我们使用了中断时间序列、倾向分数和三个独立数据源。四个变量的覆盖率变化得到了评估：妇女在医疗机构分娩、1至59个月孩子获得腹泻口服补液盐、治疗肺炎的抗生素和治疗疟疾的青蒿素。我们在生命挽救工具中使用几个场景模仿覆盖变化对死亡率的影响。

**结果:**

所有变量的覆盖都有增加，然而，肺炎抗生素在统计上没有显著增加。估计死亡率的影响方面，在两个地区，干预在第一年挽救约593（估计范围168-1060）名儿童的生命。5岁以下儿童死亡率从2008年每千例活产235人降低到2009年的210人（估计范围189-228）。如果全国采用类似的干预，每年可以挽救1.4-1.9万（估计范围0.4-2.8万）名儿童。所有场景孕产妇死亡率显示有适度降低。

**结论:**

在此环境中，免除用户费用增加了卫生服务的使用，可能对降低儿童死亡率发挥了作用。

## Резюме

**Цель:**

Определить воздействие отмены платы за медицинские услуги, взимаемой с беременных женщин и детей младше пяти лет, на уровень материнской и детской смертности в Буркина-Фасо.

**Методы:**

Два административных округа в регионе Сахель в сентябре 2008 года отменили плату, взимаемую с пациентов за роды в медицинском стационаре и за медицинские консультации для детей. Результаты оценили по 4 показателям: роды в медицинском стационаре, получение детьми от 1 до 59 месяцев пероральной регидратационной соли при диарее, антибиотиков при пневмонии и артемизина при малярии. Мы смоделировали воздействие изменений на уровень смертности с помощью инструмента «Спасенные жизни», используя несколько сценариев.

**Результаты:**

Результаты улучшились по всем четырем показателям, однако улучшение статистических данных по использованию антибиотиков при лечении пневмонии не было достаточно высоким. В отношении влияния на уровень смертности предпринятые меры помогли спасти жизни примерно 593 детей (оцениваемый диапазон 168–1060) в обоих округах в течение одного года. Это позволило понизить предполагаемый уровень смертности детей в возрасте младше 5 лет с 235 смертей на 1000 рожденных живыми в 2008 году до 210 (оцениваемый диапазон 189–228) в 2009 году. Если предпринять аналогичные меры по всей стране, это позволит сохранить от 14 000 до 19 000 детских жизней в год (оцениваемый диапазон 4000–28 000). Показатели материнской смертности понизились незначительно во всех рассматриваемых сценариях.

**Вывод:**

В данной ситуации отмена платы, взимаемой с пациентов, позволила повысить уровень пользования медицинскими услугами и способствовала сокращению детской смертности.

## Introduction

Direct charges for health care at the point of use (user fees), can limit access to health services and push households into poverty.^1^ Many governments nonetheless continue to rely heavily on user charges to finance health systems. This is especially so in low-income countries, where the health sector is usually under funded.^1^ A recent survey of 50 high-mortality countries in Africa and Asia found that 44 of these countries continue to levy user fees for health care.^2^

User fees may also hinder the achievement of the health-related Millennium Development Goals (MDGs). Of the 75 countries where more than 95% of all maternal and child deaths occur, only 23 are on track to achieve MDG 4 (reduce the mortality rate by two thirds among children less than five years of age) and only nine are on track to achieve MDG 5 (reduce the maternal mortality ratio by three quarters).^3^

Eliminating user fees for pregnant women and children has been suggested as a strategy to increase coverage of high-impact interventions to achieve the MDGs and to move towards universal health coverage.^4^^–^^6^ In 2010, the African Union called for the elimination of user fees for children less than five years of age,^7^ and several African countries have now adopted this approach.^2^

Burkina Faso is a low-income country, with 44% of the population living in poverty.^8^ In its Sahel region in 2008, user fees were eliminated for pregnant women and for children less than five years of age, in two out of the region’s four health districts.^9^ This was achieved by a complex strategy building on existing programmes to cover health-financing gaps.

To evaluate the estimated effect of eliminating user fees on the target populations we asked two questions: For key maternal and child health interventions, did the user-fee elimination strategy increase receipt of needed health services (coverage)? Consequently, did it reduce mortality in the target populations? We used analytical techniques for ex-post impact evaluation and mathematical modelling to test whether the user-fee elimination strategy increased health-care coverage and reduced mortality. We also explored the anticipated mortality impact of scaling up a similar intervention to the entire Sahel region and to the national level.

## Methods

This study was approved by the research ethics committee of the Centre de Recherche du Centre Hospitalier de l’Université de Montréal (project 09.122), Burkina Faso’s ministry of health and local ethics committees.

To assess whether eliminating user fees would affect child and maternal mortality, we triangulated evidence using multiple data sources and analytical techniques to assess the effect of the user-fee elimination programme on coverage of specific health interventions. We then used the Lives Saved Tool (LiST) to link evidence on coverage improvements to anticipated mortality declines. The user-fee elimination programme provided a subsidy to render medical services free-of-charge at point-of-care for all pregnant women and for children less than five years of age in two health districts: Dori and Sebba.^9^ Since 2007, the government organized an 80% emergency obstetrical and neonatal care (*Soins obstétricaux et néonataux d’urgence*) subsidy associated with facility-based delivery. For pregnant women, the user-fee elimination programme hence reimbursed the complementary 20%. For children, the government provides basic preventive interventions free-of-charge, but requires payment for curative services and drugs. The user-fee elimination programme reimbursed designated primary health centres to render all consultations and prescriptions free at point-of-care for children less than five years of age.^9^

The Humanitarian Aid Office of the European Commission provided funding. Regional health authorities and the German nongovernmental organization Hilfe zur Selbsthilfe were the implementation partners.

### Mathematical modelling

We modelled the effect of eliminating user fees using the freely available mathematical model LiST, version 4.53 (Johns Hopkins Bloomberg School of Public Health, Baltimore, United States of America; available from: http://www.jhsph.edu/departments/international-health/centers-and-institutes/institute-for-international-programs/list/index.html). The model structure has been described elsewhere.^10^

LiST was chosen because its target populations are identical to those of the study, and the study’s health interventions (i.e. given birth at health facility and children treated for diarrhoea, malaria or pneumonia) are represented in LiST. In addition, the model has been shown to provide accurate predictions of neonatal and child mortality associated with intervention scale-up in diverse geographical settings,^11^^–^^13^ including western Africa.^12^

#### Model parameters

We used LiST default demography, proportional mortality and intervention effectiveness estimates. All other variables were classified into one of two categories.

Category 1 consisted of variables unaffected by user-fee elimination. We updated LiST parameters using data from household surveys,^8^^,^^14^^–^^19^ United Nations agencies’ estimates,^20^^,^^21^ national administrative data^22^^,^^23^ and research reports.^24^ To ensure accurate estimation of mortality, category 1 indicators reflect changes in health-care coverage over the study period. (Appendix A, available from: http://www.equitesante.org/helpburkina/articles-scientifiques/user-fee-elimination-reduces-maternal-child-mortality). Variables potentially affected by user-fee elimination, but for which data on coverage impact were lacking, were also grouped in this category.

Category 2 consisted of four health care variables potentially affected by user-fee elimination: one related to pregnant women (giving birth at a health facility) and three related to children (treatment for diarrhoea, pneumonia or malaria, with oral rehydration salts, antibiotics or artemesinin, respectively). For these four variables, we developed statistical models – adjusted for potential confounding variables – to estimate the impact of user-fee elimination on health-care coverage. Coverage data for 2008 were taken from external sources (Table 1).^15^^,^^25^^,^^26^

**Table 1:** Definition of study variables and baseline estimates of health-care coverage, Burkina Faso, 2008

Variable	LiST variable definition^a^	Health-care coverage, % and reference	
		Sahel	National
Facility delivery	Percentage of infants delivered in a health facility	35^15^	39^15^^,^^25^
Oral rehydration salts for diarrhoea	Percentage of children with diarrhoea given sachets of oral rehydration salts	10^15^	21^25^
Antibiotics for pneumonia^b^	Proportion of children 1–59 months with suspected pneumonia or acute respiratory infection treated with antibiotics	33^c,^^26^	31^25^
Artemesinin for malaria	Proportion of children 0–59 months with a fever receiving any appropriate antimalarial	26^15^	41^25^

LiST: Lives Saved Tool.

^a^ Variables are defined as recommended for the use of the LiST model. Variables definitions correspond to data routinely collected in demographic and health surveys; the exception is receipt of antibiotics for pneumonia which is difficult to assess using household survey data.^27^

^b^ For the Sahel, as primary data were not available, we used the overall midpoint data derived from a representative survey of 16 Sahel districts in a recent evaluation of a programme to improve maternal and child health in Burkina Faso. These data were collected specifically for the LiST model using appropriate definitions. We also considered a range representing the midpoints from all 16 districts.^26^

^c^ The range for antibiotic treatment for pneumonia was 10–56%.^26^

#### Statistical modelling

For each of the variables in category 2, the intervention effect from 2008 to 2009 reflects the difference in the proportion of individuals seeking medical care due to elimination of user fees. For the three variables related to children´s health, this was due directly to user-fee elimination. For pregnant women giving birth in a facility, the difference is due to the combined effect of the user-fee elimination programme and the governmental emergency obstetrical and neonatal care subsidy.

For pregnant women giving birth in a facility, we ran interrupted time-series analyses covering a 96-month period (January 2004 to December 2011), including three years before the introduction of the emergency obstetrical and neonatal care subsidy. Population-level information was extracted from administrative records contained in Burkina Faso’s national health information database for all 71 primary health centres in all four Sahel health districts, two in which user fees were eliminated (Dori and Sebba) and two which maintained user fees (Gorom-Gorom and Djibo) but had similar health and living standards, geography, culture and climate.

We developed multilevel Poisson models (district, primary health centre, monthly deliveries) to study the effect of the emergency obstetrical and neonatal care subsidy and user-fee elimination programme both individually and in combination on the likelihood of delivery in a primary health centre. We used sitewise random intercepts and slopes and estimated intervention effects as rate ratios.^28^ In total, 6302 deliveries were recorded. Rate ratios (RR) and 95% confidence intervals (CI) were estimated by multilevel Poisson regression, adjusting for district, primary health care centres, secular trend, seasonal variation, statistical overdispersion and (via an offset) population growth.

To evaluate the effect of eliminating user fees for childhood consultations for diarrhoea, pneumonia and malaria, we used data from a household survey in the two health districts where the user fees had been eliminated.^9^^,^^29^ The sampling approach was based on probability proportional to size selection of census tracts and random selection of households.^30^ The same households were surveyed in July and August 2008 before the intervention, and again one year after implementation (July and August 2009). Survey data included households’ social and economic characteristics, health symptoms and needs within the last 30 days for children less than five years of age and health-care utilization. We developed multilevel (district, primary health centre, household) logistic regression models to estimate the effect of eliminating user fees on consultations for children reporting symptoms of diarrhoea, cough and fever.

The final regression model predicting childhood consultations included a propensity score term to adjust for overt selection biases while reducing the covariates to a one-dimensional score.^31^^,^^32^ The dependent variable for the propensity score model was the probability of an observation belonging to the intervention year 2009. The final model also contained interaction terms distinguishing the clinical subgroups – diarrhoea, cough and fever. The 12-month intervention effects for Dori were used in the LiST analysis to represent the intervention effect on diarrhoea, pneumonia and malaria coverage at one year. Odds ratios (ORs) and their CIs were applied to different scenarios of baseline risks before conversion to approximate relative risks for use in LiST modelling, because the assumption of homogeneity over varying baseline risks holds much better for ORs than for risk ratios.^33^Supplementary analyses with the variables any childhood consultation or severe diarrhoea were used to validate the main analysis. Severe diarrhoea was defined as an episode reported as life-threatening or incapacitating.

We used Stata 12 (StataCorp LP, College Station, USA) and SAS 9.3 (SAS Institute, Cary, USA) for the analyses.

#### LiST analyses

For each variable, to calculate health care coverage for 2009, we multiplied 2008 coverage values in Table 1 by estimated intervention effects from the main analyses in Table 2. We then used LiST to model the mortality impact of the difference in coverage between 2008 and 2009 due to the user-fee elimination. We projected results for the two Sahel health districts constituting the user-fee elimination target population and for the entire Sahel. We also projected the impact of scale-up to the national level in Burkina Faso. National projections apply estimated intervention effects from the main analyses in Table 2 to models parameterized to reflect national demography, epidemiology and intervention coverage. Proportional mortality and intervention effectiveness remained unchanged from analyses for the Sahel.

**Table 2:** Estimates of the effect of user-fee elimination on medical consultations used in mortality projections,^a^ in the Sahel region of Burkina Faso 2008–2009

Indicator	Statistical analysis	Control group	Data source	Intervention effect, midpoint of relative risk (95% CI)^b^
**Main analyses^c^**				
Facility delivery^d^	Interrupted time series	Yes	Administrative data from the national health information system for Dori and Sebba (districts that eliminated user fees) and Gorom Gorom and Djibo (districts that maintained user fees), before and after interventions	1.64 (1.52–1.71)
Oral rehydration salts for diarrhoea	Propensity score	No	Baseline and endline household surveys on user-fee elimination^6^	1.65 (1.09–1.98)
Antibiotics for pneumonia	Propensity score	No	Baseline and endline household surveys on user-fee elimination^6^	1.59 (0.67–2.15)
Artemesinin for malaria	Propensity score	No	Baseline and endline household surveys on user-fee elimination^6^	1.75 (1.17–2.12)
**Validating analyses^e^**				
Any childhood consultations^f^	Interrupted time series	Yes	Administrative records from a stratified random sample of primary health centres drawn from Dori (district that eliminated user fees) and Djibo (a neighbouring district that maintained user fees)	2.18 (2.17–2.18)
Severe diarrhoea^g^	Propensity score	No	Baseline and endline household surveys on user-fee elimination	1.88 (1.37–2.10)

CI: confidence interval.

^a^ All analyses used multilevel regression models adjusted for confounding variables to compare service use before introducing the interventions and at 12 months after user-fee elimination.

^b^ Values are for Dori, because they represent a more conservative intervention effect.

^c^ These results were used in all mortality projections.

^d^ For facility delivery, the intervention effect represents the combined effect at 12 months of the emergency obstetrical and neonatal care) subsidy (*Soins obstétricaux et néonataux d’urgence)* and the user-fee elimination intervention. The analysis includes data from three years before the start of the two interventions. ^e^ Used to validate results from the main analyses. Results were not used in mortality projections.

^f^ This analysis considers childhood consultations for any indication. The sample included 12 primary health centres in Dori (district with user-fee elimination) and six primary health centres in Djibo (district withoutuser-fee elimination).^29^

^g^ We did a propensity score analysis to investigate the hypothesis that the intervention effect would be greater in those with more severe diarrhoea, defined as those in which a child’s activities were restricted or life was endangered.

Sensitivity analyses were used to explore the consequences of a range of reasonable alternatives. As LiST does not permit probabilistic sensitivity analyses, we focused on univariate and scenario-based approaches. Scenarios considered uncertainty in values for key model parameters as well as statistical uncertainty when estimating intervention effects (Appendix B; available from: http://www.equitesante.org/helpburkina/articles-scientifiques/user-fee-elimination-reduces-maternal-child-mortality).

For the Sahel, we had no reliable data on existing levels of antibiotic coverage for pneumonia in 2008. We explored three scenarios representing average, low and high estimates of antibiotic coverage for children. Within each scenario, we estimated lives saved based on the midpoint, and the upper and lower 95% CIs for user-fee elimination intervention effects. The Sahel mortality scenario is based on Burkina Faso’s 2010 demographic and health survey and multiple indicator cluster survey, which estimated 235 deaths per 1000 live births for under-five mortality.^15^ Estimated Sahel maternal mortality was 840 per 100 000 births.^34^

National-level analyses considered high- and low-mortality scenarios due to uncertainty concerning the background of mortality rates. High-mortality scenario for children less than five years of age was based on United Nations Inter-agency Group for Child Mortality Estimation, which was 178 deaths per 1000 live births for Burkina Faso in 2010,^35^ similar to the 2008 estimates of the Institute for Health Metrics and Evaluation (164.7 deaths; 95% CI: 140.4–190.9).^36^ For maternal mortality, we based the high mortality scenario on the values given in LiST, 700 deaths per 100 000 births.^25^

The low-mortality scenario is based on Burkina Faso’s 2010 demographic and health survey and multiple indicator cluster survey, which recorded 129 deaths per 1000 live births for under-five mortality and 307 deaths per 100 000 birth for maternal mortality.^15^ For both scenarios, we calculated lives-saved estimates based on the midpoint, and the upper and lower 95% CIs for user-fee elimination intervention effects. Appendix B provides further details.

## Results

The interrupted time series analysis showed that the likelihood that pregnant women would give birth at a health facility significantly increased at all time-points analysed after user fees had been eliminated. A year after elimination of user charges, the relative risk of delivery in a health facility was 1.80 (95% CI: 1.46–2.20) in Dori and 1.96 (95% CI: 1.50–2.00) in Sebba when compared with the pre-intervention period (Table 3).

**Table 3:** Likelihood of a pregnant women delivering at a health facility after user-fee elimination,^a^ Dori and Sebba health districts in Burkina Faso, September 2008–September 2011

Months after elimination	RR (95% CI)^b^ by health district	
	Dori	Sebba
1	1.72 (1.43–2.06)	1.94 (1.53–2.44)
6	1.75 (1.45–2.12)	1.94 (1.52–2.48)
12	1.80 (1.46–2.20)^c^	1.96 (1.50–2.00)
18	1.84 (1.47–2.30)	1.97 (1.48–2.63)
24	1.88 (1.47–2.40)	1.98 (1.44–2.73)
30	1.93 (1.47–2.53)	2.00 (1.41–2.84)
36	1.98 (1.47–2.66)	2.01 (1.37–2.96)

CI: confidence interval; RR: rate ratio.

^a^ Total user-fee exemptions is a combination of the emergency obstetrical and neonatal care (*Soins obstétricaux et néonataux d’urgence*) subsidy and the user-fee elimination intervention.

^b^ Adjusted for district, primary health care centres, secular trend, seasonal variation, over-dispersion and population growth.

^c^ The 12-months intervention effect for Dori was used in the modelling analysis (Table 2) to represent the intervention effect on facility deliveries at one year.

Table 4 summarizes the household survey sample used to study the changes in medical consultations for children less than five years of age. Between 2008 and 2009, there was a 1.5-fold and 2.5-fold increase in all-cause consultations in Dori and Sebba, respectively.

**Table 4:** Characteristics of the household survey sample used to estimate the effect of user-fee elimination on malaria, pneumonia and diarrhoea treatment for children less than five years of age, Dori and Sebba health districts in Burkina Faso, 2008–2009

Characteristic	Dori			Sebba		
	2008	2009		2008	2009	
**Household**						
Surveyed per year, no.	1 257	1 098		831	755	
Surveyed both years,^a^ no.	1 069			713		
Annual household health expenditure in CFA franc, median (interquartile range)^b^	174 115 (110 380–299 750)			142 890 (86 240–241 525)		
Education of head of household, no. (%)						
None	1 159 (92.2)			802 (96.5)		
Attended or completed primary	33 (2.6)			11 (1.3)		
Attended or completed secondary	25 (2.0)			3 (0.4)		
Attended or completed senior secondary or higher	5 (0.4)			0 (0.0)		
Missing data	35(2.8)			15 (1.8)		
**Children**						
Eligible, no.	1 454	1 309		861		836
Age distribution, years, no. (%)						
< 1	305 (21.0)	298 (22.8)		180 (20.9)		172 (20.6)
1 to < 2	233 (16.0)	253 (19.3)		132 (15.3)		158 (18.9)
2 to < 3	325 (22.4)	210 (16.0)		201 (23.3)		130 (15.6)
3 to < 4	299 (20.6)	280 (21.4)		187 (21.7)		189 (22.6)
4 to < 5	292 (20.1)	268 (20.5)		161 (18.7)		187 (22.4)
**Reported illnesses episodes, no. (%)^c,d^**	238 (16.4)	185 (14.1)		100 (11.6)		73 (8.7)
Severe illness^e^	124 (52.1)	118 (63.8)		50 (50.0)		56 (76.7)
Health centre visits	85 (35.7)	102 (55.1)		28 (28.0)		51 (69.9)
Severe episodes^e^	41 (48.2)	78 (76.5)		17 (60.7)		43 (84.3)
Non-severe episodes	44 (51.8)	24 (23.5)		11 (39.3)		8 (15.7)

CFA: Communauté Financière Africaine.

^a^ Estimated attrition rate was 9.3% in Dori and 4.2% in Sebba.

^b^ Total expenses by households reporting an ill child less than five years of age. The average exchange rate over the period 2008–2009 was 510.53 CFA francs to 1 United States dollar.^37^

^c^ Illnesses that began within the recall period of 30 days before the interview.

^d^ Sebba visits occurred during the beginning of the rain season with little flooding, therefore fewer children were sick.

^e^ Defined by the respondent as life threating or severely impairing daily activities.

When analysing each clinical subgroup, results of the propensity score analysis on childhood consultations show that medical consultations for diarrhoea and probable malaria increased in each of the two user-fee elimination districts, whereas consultations for pneumonia only increased significantly in Sebba (Table 5).

**Table 5:** Estimates of the likelihood of medical care being sought for children less than five years of age after user-fee elimination, the Sahel region of Burkina Faso, 2008–2009^a,b^

Variable by health district	Estimate of the likelihood of seeking care (SE)	OR (95% CI)	Relative risk (95% CI)
**Dori**			
Fever consultations			
2009	1.026 (0.502)		
2008	−0.321 (0.338)		
Change 2008–2009	1.348 (0.541)	3.85 (1.32–11.18)	1.75 (1.17–2.12)^c^
Cough consultations			
2009	0.761 (0.703)		
2008	−0.289 (0.546)		
Change 2008–2009	1.049 (0.849)	2.86 (0.54–15.22)	1.59 (0.67–2.15)^c^
Diarrhoea consultations			
2009	1.120 (0.507)		
2008	−0.174 (0.375)		
Change 2008–2009	1.294 (0.574)	3.65 (1.18–11.30)	1.65 (1.09–1.98)^c^
**Sebba**			
Fever consultations			
2009	2.235 (0.663)		
2008	−0.068 (0.522)		
Change 2008–2009	2.303 (0.713)	10.01 (2.46–40.75)	1.87 (1.44–2.02)
Cough consultations			
2009	1.928 (0.813)		
2008	−0.096 (0.627)		
Change 2008–2009	2.024 (0.927)	7.57 (1.22–46.99)	1.83 (1.10–2.05)
Diarrhoea consultations			
2009	2.160 (0.660)		
2008	−0.061 (0.519)		
Change 2008–2009	2.221 (0.713)	9.21 (2.26–37.56)	1.85 (1.40–2.01)

CI: confidence interval; LiST: Lives Saved Tool; OR: odds ratio; SE: standard error.

^a^ Represents fixed effects estimates from a multilevel logistic regression model. Of the 1782 households with data in both survey waves, 270 reported childhood consultations.

^b^ A propensity score term was included in the final model to adjust for confounding on observed variables. Propensity score estimation included the following covariates: age of child, identity of survey respondent, age of head of household, ongoing illness versus completed, duration of illness, and previously reported illness in 2008.

^c^ Value used in the LiST analysis to represent the intervention effect on coverage at one year.

Table 2 provides intervention effect estimates used for the mortality projections. The estimates showed that the likelihood of using health services by those in need increased in the user-fee elimination groups. In Dori, the midpoint relative risk estimates on coverage ranged from 1.6 to 2.2 across all six indicators (Table 2). For all indicators, results for the two intervention districts were similar but the effect was slightly greater for Sebba (Table 3 and Table 5). As the results from Dori provided a more conservative estimate of the intervention effect, those values were used in LiST modelling (Table 2).

We estimated that eliminating user fees saved an average of 593 lives of children less than five years of age (estimate range: 168–1060 lives) in the study population during the first year of the user-fee elimination programme. Eliminating user fees throughout the Sahel region would save an average of 1350 lives of children less than five years of age (estimate range 383–2414 lives) and reduce child mortality by an estimated 11% (estimate range 3–20%); from 235 per 1000 live births in 2008^15^ to 210 per 1000 live births in 2009 (estimate range 189–228) (Table 6). Delivery in a health facility had a modest estimated impact on maternal mortality. Model projections show that 40 (95% CI: 29–51) women’s lives were saved in the study population, and that 91 (95% CI: 67–117) would be saved if a similar intervention were introduced throughout the Sahel region.

**Table 6:** Projection of lives saved after user-fee elimination in children less than five years of age in the study districts, and projection for the entire Sahel region of Burkina Faso, 2008–2009

Scenario^a^	Dori and Sebba districts,^b^ midpoint number of lives saved (95% CI)	Sahel region		
		Midpoint number of lives saved (95% CI)	Mortality rate 2009 (95% CI)	% reduction in mortality between 2008^c^ and 2009 (95% CI)
Average	593 (180–921)	1350 (409–2 097)	210 (195–227)	11 (4–17)
Low	482 (168–699)	1098 (383–1 593)	215 (205–228)	9 (3–13)
High	772 (168–1 060)	1758 (383–2 414)	201 (189–228)	15 (3–20)

CI: confidence interval.

^a^ Mortality scenarios were defined based on uncertainty in coverage values for the child health indicator “receipt of antibiotics for pneumonia in 2008”. The average scenario is based on the overall midpoint from a representative survey of 16 districts in a recent evaluation of a programme to improve maternal and child health in Burkina Faso; low and high scenarios reflect the highest and lowest midpoints from the 16 districts.^26^

^b^ The study districts represent 44% of the Sahel population.

^c^ In 2008, the mortality rate was 235 children less than five years of age per 1000 live births in the Sahel.^15^

When simulating a nationwide user-fee elimination, the projected under-five mortality was reduced by 16% (95% CI: 4–26) in the high-mortality scenario and 17% (95% CI: 4–26) in the low-mortality scenario (Table 7).

**Table 7:** Projection of child and maternal lives saved after user-fee elimination in Burkina Faso, 2008–2009

Scenario	Children less than five years of age				Mothers	
	Midpoint number of lives saved (95% CI)	Mortality rate 2009 (95% CI)	% reduction in mortality between 2008 and 2009 (95% CI)		Midpoint number of lives saved (95% CI)	Mortality rate 2009 (95% CI)
Low	14 183 (3719–20 451)	107 (97–124)	17 (4–26)		489 (366–637)	232 (210–251)
High	19 200 (5070–29 892)	148 (131–169)	16 (4–26)		1123 (880–1456)	529 (429–572)

CI: confidence interval.

Notes: Scenarios were defined based on differences in estimated mortality rates. In Burkina Faso 2008, for the low mortality scenario the child mortality rate was 129 children less than five years of age per 1000 live births^15^ and the maternal mortality ratio was 307 per 100 000 live births.^15^ For the high mortality scenario, the child mortality rate was 178 children less than five years of age per 1000 live births^25^^,^^35^ and the maternal mortality ratio was 700 per 100 000 live births.^25^

The main sources of the modelled mortality in children were malaria, diarrhoea, pneumonia and neonatal causes. The main health interventions for projected under-five lives saved were anti-malarials, antibiotics for pneumonia, oral rehydration salts for diarrhoea, labour and delivery management, antenatal corticosteroids for preterm labour, and neonatal resuscitation. The latter three interventions, associated with delivery in a health facility, reduced neonatal mortality. Other interventions contained in the LiST model reduced mortality by 5% or less (Table 8).

**Table 8:** Projected reduction in mortality by intervention for children less than five years of age, in the Sahel region of Burkina Faso, 2008–2009

Intervention	Mean reduction, % (range)
Antimalarials: Artemesinin compounds for malaria	34 (24–50)
Oral antibiotics: case management of pneumonia	18 (0–43)
Oral rehydration solution	9 (5–14)
Labour and delivery management	13 (5–25
Antenatal corticosteroids for preterm labour	8 (4–15)
Neonatal resuscitation	4 (2–7)
Clean birth practices	3 (1–5)
Antibiotics for preterm premature rupture of membranes	2 (1–4)
Improved water source	2 (2–5)
Immediate assessment and stimulation	2 (1–4)
Hand washing with soap	2 (1–3)
Clean postnatal practices	1 (0–2
Thermal care	0 (0–1)
Syphilis detection and treatment	0 (0–0)
Promotion of breastfeeding	0 (0–0)
**Total^a^**	**100**

^a^ Inconsistencies arise due to rounding.

Note: All intervention definitions from LiST.^25^^,^^27^

## Discussion

Previous studies have shown that when user charges are abolished, the use of health services tends to increase. However, it has been unclear whether this increase reflects appropriate use of health services and evidence for improved health is lacking.^38^ Our findings show that a complex intervention based on eliminating user fees for pregnant women and children increased intervention coverage. Multiple independent statistical analyses exploiting distinct analytical methods and data sources confirmed that service coverage was approximately one and a half to two times higher in the districts where user fees had been eliminated, as compared with the comparison districts. Model-based projections for a single year show that coverage increases are likely to have brought about substantial reductions in neonatal and child mortality, and a modest reduction in maternal mortality. As the intervention was carried out in a region with poor health indicators, the estimated reductions in mortality are therefore particularly important.

Strengths of the study include the following: the use of a validated model to project mortality impact; availability of high-quality data from a study designed specifically to measure the user-fee elimination effect; employment of appropriate study designs and statistical methods to quantify the intervention impact while controlling for potential confounding variables and selection biases; exploitation of multiple data sources and statistical techniques to enable cross-checking of results and convergence on reasonable values for intervention effects; and a conservative approach to estimating mortality impact.

The modelling approach was conservative in several ways. First, numerous health interventions affected by the user-fee elimination programme could not be modelled due to lack of data – for example, antenatal care visits, childhood vaccinations, breastfeeding promotion, antibiotics for dysentery, zinc for diarrhoea and vitamin A for measles. Second, the analyses used estimates from the health district with the lower intervention effect. Third, time-series analyses for facility deliveries show increasing intervention effects over time not captured in this one-year analysis. Fourth, other analyses show a stronger intervention effect in those at higher mortality risk.

There were several study limitations. First, mortality projections were based on mathematical modelling. We lessened the risk of error by using a validated model to estimate mortality with conservative assumptions and extensive sensitivity analyses. Second, for some key parameters, there is uncertainty regarding baseline levels. We developed scenarios reflecting a range of reasonable values from the strongest available data sources. Third, we were unable to consider statistical uncertainty associated with parameter estimates from the demographic and health survey data,^15^ as only average values were available. This limitation is common to all LiST analyses and likely to have a non-systematic impact on mortality projections. Fourth, we focused on overall effects. A separate analysis demonstrated that the intervention benefitted the poor.^29^ Fifth, increasing consultations does not guarantee appropriate treatment and eliminating user fees can have potential negative effects on service quality.^38^^,^^39^ Fifth, during the user-fee elimination it was shown that the quality of prescriptions was maintained.^40^ Insofar as the quality of care was similar before and after the intervention, effectiveness remains constant and therefore the conclusion that lives would be saved holds valid. However, inappropriate treatment practices would reduce the estimates of absolute numbers of lives saved. Sixth, while convergence of findings from multiple data sources and methods promises good internal validity, generalization of results to the national level requires evidence of external validity which is currently lacking. We therefore recommend that national projection estimates should be seen as exploratory.

Research on interventions to improve the delivery, practice and organization of health-care services poses challenges. Mortality averted by such interventions cannot be directly observed and an individual or cluster randomized trial of proven interventions with a mortality endpoint is not likely to be performed for reasons of ethics and feasibility. Under the circumstances, judicious synthesis of observational evidence using rigorous statistical techniques, multiple data sources and mathematical modelling is a suitable approach to answer an important policy question. Conclusions should be interpreted with appropriate caution.

Three published studies have examined the estimated impact of eliminating user fees on mortality. A mathematical modelling study explored the impact of a hypothetical user-fee elimination programme in 20 African countries and concluded that eliminating user fees for pregnant women and children could contribute to saving lives.^5^ A case study used LiST to illustrate how a range of government policies in the Niger, including provision of free health care for women and children, decreased child mortality.^41^ An econometric analysis of Thailand’s “30-baht” health-care reform, which sharply increased funding for hospitals while reducing co-payments, found that the programme increased access to health care among the poor, reduced infant mortality and equalized disparities in infant mortality.^42^ These three studies are in line with our conclusions that the abolition of user fees has lifesaving potential. However, the data used in the modelling study^5^ to estimate intervention effects are dated, and rely on studies from several countries and programmes with differing degrees of controls for confounding factors. The studies in the Niger^41^ and Thailand^42^ traced observed results of an ensemble of policies and did not specifically address the question of user-fee elimination.

A single randomized controlled trial in Ghana provides the only direct evidence of the impact of removing out-of-pocket payments on health outcomes in developing countries.^43^ It shows that in this study setting, eliminating user fees had an effect on care-seeking behaviour but not on the measured health outcomes. Possible explanations for this lack of effect include limited statistical power, failure to remove other barriers to service use, the possibility that the increase in using the intervention was too small to produce a clear effect on health, and residual confounding factors.^43^^,^^44^ Results nevertheless demonstrate the value of measuring health endpoints in addition to those relating to service use.

Most low- and middle-income countries are unlikely to achieve MDGs 4 and 5 by the 2015 target date.^3^^,^^35^^,^^45^ Alongside other strategies targeting non-financial barriers to access,^46^ our findings demonstrate that well designed policies to eliminate user fees can contribute to attainment of MDGs 4 and 5 and advance universal health coverage.

The most important question now concerns whether these results can be replicated at scale. Although embedded in the existing health system, this instance of user-fee elimination was implemented in a specific locale with the help of a nongovernmental organization. To date, most user-fee elimination policies led by national governments in western Africa have faced implementation difficulties. In the light of the existing evidence and study design challenges, as the next step, we recommend that user-fee elimination should be introduced on a wider scale, accompanied by rigorous evaluation. Effective scale-up will require careful policy design to ensure that solid financing mechanisms are in place,^39^ and reduce potential problems related to weak health systems, including deficiencies in human resources management, quality of care, supply chain logistics, and informal payments.^19^

## References

[1] World health report 2010 – Health systems financing: the path to universal coverage. Geneva: World Health Organization; 2010. 10.2471/BLT.10.078741 PMC2878164 20539847

[2] Witter S. Mapping user fees for health care in high-mortality countries: evidence from a recent survey. London: HLSP Institute; 2010.

[3] Building a future for women and children: the 2012 report. Washington: Countdown to 2015; 2012. Available from: http://www.countdown2015mnch.org/reports-and-articles/2012-report [cited 2014 May 9].

[4] Yates R. Women and children first: an appropriate first step towards universal coverage. Bull World Health Organ. 2010;88(6):474–5. 10.2471/BLT.09.074401 20539868 PMC2878151

[5] James C, Morris SS, Keith R, Taylor A. Impact on child mortality of removing user fees: simulation model. BMJ. 2005;331(7519):747–9. 10.1136/bmj.331.7519.747 16195292 PMC1239978

[6] Address inequities [Partnership for Maternal Newborn and Child Health Knowledge Summary No. 9]. Geneva: World Health Organization; 2010. Available from: www.who.int/pmnch/knowledge/publications/summaries/ks9.pdf?ua=1 [cited 2014 Jul 7].

[7] Actions on maternal, newborn and child health and development in Africa by 2015. In: Assembly of the African Union: Fifteenth Ordinary Session; 2010 July 27; Kampala, Uganda. Addis Ababa: African Union; 2010. Available from: http://www.au.int/en/sites/default/files/ASSEMBLY_EN_25_27_July_2010_BCP_ASSEMBLY_OF_THE_AFRICAN_UNION_Fifteenth_Ordinary_Session.pdf [cited 2014 Jul 7].

[8] Enquête Intégrale sur les Conditions de Vie des Ménages. Ouagadougou: Institut National de la Statistique et de la Démographie; 2009. French.

[9] Ridde V, Queuille L, Atchessi N, Samb O, Heinmüller R, Haddad S. The evaluation of an experiment in healthcare user fees exemption for vulnerable groups in Burkina Faso. Field Actions Sci Rep [Internet]. 2013; Special issue 8. Available from: http://factsreports.revues.org/1758 [cited 2014 Aug 26].

[10] Winfrey W, McKinnon R, Stover J. Methods used in the Lives Saved Tool (LiST). BMC Public Health. 2011;11 Suppl 3:S32. 10.1186/1471-2458-11-S3-S32 21501451 PMC3231906

[11] Friberg IK, Bhutta ZA, Darmstadt GL, Bang A, Cousens S, Baqui AH, et al. Comparing modelled predictions of neonatal mortality impacts using LiST with observed results of community-based intervention trials in South Asia. Int J Epidemiol. 2010;39 Suppl 1:i11–20. 10.1093/ije/dyq017 20348113 PMC2845856

[12] Hazel E, Gilroy K, Friberg I, Black RE, Bryce J, Jones G. Comparing modelled to measured mortality reductions: applying the Lives Saved Tool to evaluation data from the Accelerated Child Survival Programme in West Africa. Int J Epidemiol. 2010;39 Suppl 1:i32–9. 10.1093/ije/dyq019 20348124 PMC2845858

[13] Ricca J, Prosnitz D, Perry H, Edward A, Morrow M, Ernst P, et al. Comparing estimates of child mortality reduction modelled in LiST with pregnancy history survey data for a community-based NGO project in Mozambique. BMC Public Health. 2011;11 Suppl 3:S35. 10.1186/1471-2458-11-S3-S35 21501454 PMC3231909

[14] Etat et structure de la population. Ouagadougou: Institut National de la Statistique et de la Démographie; 2009 French.

[15] Enquête Démographique et de Santé et à Indicateurs Multiples (EDSBF-MICS IV) de 2010: Rapport final. Ouagadougou: Institut National de la Statistique et de la Démographie; 2012 French.

[16] Enquête Démographique et de Santé de 2003: Rapport final. Ouagadougou: Institut National de la Statistique et de la Démographie; 2004 French.

[17] Natalité-Fécondité. Ouagadougou: Institut National de la Statistique et de la Démographie; 2009 French.

[18] Analyse des résultats de l'enquête annuelle sur les conditions de vie des ménages en 2007. Ouagadougou: Institut National de la Statistique et de la Démographie; 2007 French.

[19] Xu K, Evans DB, Kadama P, Nabyonga J, Ogwal PO, Nabukhonzo P, et al. Understanding the impact of eliminating user fees: utilization and catastrophic health expenditures in Uganda. Soc Sci Med. 2006;62(4):866–76. 10.1016/j.socscimed.2005.07.004 16139936

[20] Joint Monitoring Programme (JMP) for Water Supply and Sanitation. Burkina Faso: estimates on the use of water sources and sanitation facilities (1980–2012) [Internet]. Geneva and New York: World Health Organization and United Nations Children’s Fund; 2014. Available from: http://www.wssinfo.org/documents/?tx_displaycontroller[type]=country_files/ [cited 2014 Aug 26].

[21] WHO vaccine-preventable diseases: monitoring system, 2010 global summary. Geneva: World Health Organization; 2010.

[22] Direction Générale de l'Information et des Statistiques Sanitaires. Tableau de bord santé 2010. Ouagadougou: Ministère de la Santé, Burkina Faso; 2011. [French. ]

[23] Synthèse des indicateurs pour l'année 2010. Ouagadougou: Ministère de la Santé, Burkina Faso: 2010 French.

[24] Cousens S, Blencowe H, Stanton C, Chou D, Ahmed S, Steinhardt L, et al. National, regional, and worldwide estimates of stillbirth rates in 2009 with trends since 1995: a systematic analysis. Lancet. 2011;377(9774):1319–30. 10.1016/S0140-6736(10)62310-0 21496917

[25] LiST: The Lives Saved Tool, version 4.53. [Internet]. Baltimore: Johns Hopkins Bloomberg School of Public Health; 2013. Available from: http://www.jhsph.edu/departments/international-health/centers-and-institutes/institute-for-international-programs/list/index.html [cited 2014 Aug 26].

[26] Evaluation indépendante du projet d'accélération de la réduction de la mortalité maternelle, néonatale et infanto-juvénile dans les régions sanitaires du nord et centre-nord au Burkina Faso: Enquête de couverture de base - Rapport d'analyse. Ouagadougou: Institut National de la Statistique et de la Démographie; 2012 French.

[27] DeCormier Plosky W, Stover J, Winfrey B. The Lives Saved Tool: a computer program for making child survival projections. Washington: USAID and Health Policy Initiative; 2011.

[28] Snijders T, Bosker R. Multilevel analysis: an introduction to basic and advanced multilevel modelling. 2nd ed. London: SAGE Publications Limited; 2012.

[29] Ridde V, Haddad S, Heinmüller R. Improving equity by removing healthcare fees for children in Burkina Faso. J Epidemiol Community Health. 2013;67(9):751–7. 10.1136/jech-2012-202080 23776054 PMC3756435

[30] Bennett S, Woods T, Liyanage WM, Smith DL. A simplified general method for cluster-sample surveys of health in developing countries. World Health Stat Q. 1991;44(3):98–106. 1949887

[31] Imbens GW. Nonparametric estimation of average treatment effects under exogeneity: A review. Rev Econ Stat. 2004;86(1):4–29 10.1162/003465304323023651

[32] Guo S, Fraser MW. Propensity score analysis: statistical methods and applications. Thousand Oaks: SAGE Publications Ltd; 2010.

[33] Cook TD. Advanced statistics: up with odds ratios! A case for odds ratios when outcomes are common. Acad Emerg Med. 2002;9(12):1430–4. 10.1111/j.1553-2712.2002.tb01616.x 12460851

[34] Burkina Faso: child marriage worsens population pressure. New York: IRIN; 2009. Available from http://www.irinnews.org/printreport.aspx?reportid=83505 [cited 2014 Aug 19].

[35] United Nations Inter-agency Group for Child Mortality Estimation. Levels and trends in child mortality: report 2012. New York: United Nations Children’s Fund; 2012.

[36] Institute for Health Metrics and Evaluation. Child mortality estimates and MDG 4 attainment by country 1990–2011. Seattle: Institute for Health Metrics and Evaluation; 2011.

[37] Official exchange rate (LCU per US$, period average). World Development Indicators: Data [Internet]. Washington: World Bank; 2013. Available from: http://data.worldbank.org/indicator/PA.NUS.FCRF [cited 2014 June 17].

[38] Lagarde M, Palmer N. The impact of user fees on access to health services in low- and middle-income countries. Cochrane Database Syst Rev. 2011; (4):CD009094. 21491414 10.1002/14651858.CD009094 PMC10025428

[39] James CD, Hanson K, McPake B, Balabanova D, Gwatkin D, Hopwood I, et al. To retain or remove user fees?: reflections on the current debate in low- and middle-income countries. Appl Health Econ Health Policy. 2006;5(3):137–53. 10.2165/00148365-200605030-00001 17132029

[40] Atchessi N, Ridde V, Haddad S. Combining user fees exemption with training and supervision helps to maintain the quality of drug prescriptions in Burkina Faso. Health Policy Plan. 2013;28(6):606–15. 10.1093/heapol/czs100 23073891

[41] Amouzou A, Habi O, Bensaïd K; Niger Countdown Case Study Working Group. Reduction in child mortality in Niger: a Countdown to 2015 country case study. Lancet. 2012;380(9848):1169–78. 10.1016/S0140-6736(12)61376-2 22999428

[42] Gruber J, Hendren N, Townsend RM. The great equalizer: Health care access and infant mortality in Thailand. Am Econ J Appl Econ. 2014;6(1):91–107. 10.1257/app.6.1.91 24772234 PMC3998713

[43] Ansah EK, Narh-Bana S, Asiamah S, Dzordzordzi V, Biantey K, Dickson K, et al. Effect of removing direct payment for health care on utilisation and health outcomes in Ghanaian children: a randomised controlled trial. PLoS Med. 2009;6(1):e1000007. 19127975 10.1371/journal.pmed.1000007 PMC2613422

[44] Ridde V, Haddad S. Abolishing user fees in Africa. PLoS Med. 2009;6(1):e1000008. 19127976 10.1371/journal.pmed.1000008 PMC2613423

[45] Lozano R, Wang H, Foreman KJ, Rajaratnam JK, Naghavi M, Marcus JR, et al. Progress towards Millennium Development Goals 4 and 5 on maternal and child mortality: an updated systematic analysis. Lancet. 2011;378(9797):1139–65. 10.1016/S0140-6736(11)61337-8 21937100

[46] Evans DB, Hsu J, Boerma T. Universal health coverage and universal access. Bull World Health Organ. 2013;91(8):546–546A. 10.2471/BLT.13.125450 23940398 PMC3738317

